# Influence of Loading Mode on the Biaxial Stress–Strain Curve at Hydraulic Bulge Test

**DOI:** 10.3390/ma17235762

**Published:** 2024-11-25

**Authors:** Jiří Sobotka, Pavel Solfronk, Martin Švec, David Koreček

**Affiliations:** Department of Engineering Technology, Faculty of Mechanical Engineering, Technical University of Liberec, Studentská 1402/2, 46117 Liberec, Czech Republic; pavel.solfronk@tul.cz (P.S.); martin.svec@tul.cz (M.Š.); david.korecek@tul.cz (D.K.)

**Keywords:** stress–strain curve, sheet metal forming, state of stress, hydraulic bulge test, yield locus, photogrammetry, GOM Correlate Pro

## Abstract

Stress–strain curves are generally a very important material characteristic. For example, in numerical simulations, especially in sheet metal forming, stress–strain curves represent one of the most important data inputs. However, there is quite a wide range of parameters that influence their outline under the chosen technological conditions and, therefore, must always be taken into account. Among them, the influence of stress state and loading history is also relevant. In addition to that, to properly define the advanced yield conditions used in numerical simulations, it is also necessary to perform material tests under multi-axial stress states. For the above reasons, the present paper deals with the influence of the loading mode on the resulting outline of stress–strain curves under the equi-biaxial stress state at hydraulic bulge test (HBT). In light of the different loading modes, the classical continuous increase in pressure in accordance with ISO 16808 was compared with the so-called ramp test, where holding times for a duration of 90 s were applied. Two materials were selected for experiments, namely, a dual-phase steel (DP steel) with UTS of 500 MPa and an interstitial-free steel (IF steel) with a yield strength of 150 MPa. The results revealed totally different deformation behaviour of the tested materials depending on the used loading mode. Moreover, an evaluation of the microstructure was performed as well to clarify the measured results. The contactless optical system GOM Correlate Pro was used to evaluate the results of the HBT.

## 1. Introduction

In light of the proper identification and subsequent evaluation of the material deformation behaviour, so-called stress–strain curves represent a truly very important characteristic. In general, their measurement and knowledge are some of the most important parts of material testing, and that is why there are quite a lot of books and standards on how their computation and determination should be carried out, e.g., [1,2,3]. In addition to that, there are quite a lot of parameters (mainly temperature and strain rate) that can influence them. Last but not least, they also differ in light of the used stress states. Very often, the tensile test is used, so the uniaxial stress–strain curve is determined. However, a proper description of the overall material deformation behaviour, e.g., for advanced yield conditions to be used in Finite Element Analysis (FEA), also requires their determination under other states of stress. Among them, the so-called equi-biaxial state of stress is one of the most important ones. That is why, in this paper, two quite different materials (high-strength steel and deep-drawing material) were tested using the hydraulic bulge test (HBT), and biaxial stress–strain curves were determined according to standard ČSN EN ISO 16808 [4]. Moreover, different loading modes were applied at their measurement to reveal the deformation behaviour at HBT under a continuous increase in pressure as well as under the so-called ramp test, where, at the chosen pressure values, a holding time of 90 s was always applied.

As was already mentioned before, very often, biaxial stress–strain curves are required for solving different mechanical engineering problems. These are especially important in applications of the yield locus for the utilisation of FEA. Generally, almost all the theoretical aspects of FEA that are needed in the engineering industry, that is, the basic concept, math equations, mechanics of materials, and so on, are given in the work of Kim et al. [5]. Suitable examples of the application of FEA in structural mechanics are shown, e.g., in [6,7]. Regarding the focus of this paper, a numerical model to investigate the influence of different parameters (e.g., stress and strain distribution) is discussed in Reis et al. [8]. Other authors use numerical simulations mostly to incorporate different yield loci [9,10], increase the accuracy of FEA and even neural networks [11,12], or, e.g., to compare the strain distribution from numerical simulations and experiments [13]. After that, basic theoretical approaches and methods of determining stress–strain curves and constitutive equations taking into account the strain, strain rate, and temperature are described in [14,15,16,17].

Because the testing of material properties via the HBT generally means that, in light of the state of stress, there is equi-biaxial stretching (thus, biaxial stress–strain curves), there exists an effort to achieve another state of stress. Very often, the so-called elliptical HBT is used, so the plain strain is achieved [18,19,20,21]. Moreover, a modified Nakajima test without inverse parameter identification was proposed by Eder et al. [22] to increase the accuracy of the given equi-biaxial flow curves. Very important research in this branch is about testing the influence of used hardening models, e.g., Lezen et al. [23] used the HBT to characterise kinematic hardening under nonlinear strain paths. The final results revealed higher accuracy than the data from the cyclic tensile–compression tests. Sometimes, instead of the HBT, cruciform specimens are used, which also makes it possible to achieve biaxial loading (and sometimes unloading is required as well) of specimens [24,25]. In addition to that, the HBT can be used to evaluate, e.g., fracture [26,27] or even fatigue [28] characteristics.

Quite a lot of interest in this area of testing is also devoted to the influence of temperature on stress–strain curves. For example, Mulder et al. [29] proposed the analytical temperature compensation model with respect to the flow stress. Boyer [30,31] studied and already proposed a new testing device, which enables the HBT to be carried out at high temperatures. The influence of higher temperatures on the effective stress–strain behaviour and modified Johnson–Cook model for strain hardening was developed by Ashrafian and Hosseini [32]. Some authors also studied the effect of warm forming temperatures or elevated temperatures on the flow curves [33,34,35].

The influence of different strain rates on the biaxial stress–strain curves has also been of great interest in recent years. Jocham et al. [36] investigated the strain rate sensitivity of material DC06 both at HBT and tensile test. Suttner and Merklein [37] applied the constant strain rate at hydraulic bulge test for different materials. From the strain rate point of view, great research interest is also devoted to the application of quite high strain rates [38,39,40,41], sometimes also for the pneumatic bulge test [42]. However, regarding the application of different loading modes, already with the holding times (or just keeping constant stress), there are just a few articles [43,44], but these are about testing the superplastic materials.

Additionally, the lack of knowledge about the deformation behaviour of common materials used in car-body design under the equi-biaxial stress state depending on the different loading modes was the major reason to carry out the study described in this paper. In the previously mentioned studies, the right holding times at the relevant pressure values were not applied during the HBT; thus, their influence on the final position of the stress–strain curves was not monitored. Furthermore, because these results are especially important, e.g., for the proper computation of the yield locus or just for FEA, two commonly used materials (high-strength steel as well as deep-drawing material) were selected to be tested at HBT. That is why, besides the classical measuring of HBT performed in accordance with ISO 16808, also, so-called ramp tests at HBT were carried out, where, for every integer multiple of actual pressure values, a holding time of a duration of 90 s was applied. Generally, the major aim of this paper was to find out if there is such an influence of different loading modes, especially in light of the final comparison between continuous “classical” measurement and ramp tests. In addition to that, there was also the possibility to reveal the influence of the own magnitude of holding time on the final position of stress–strain curves. Moreover, there was also a presumption that the evaluation of microstructure with the help of scanning electron microscopy (SEM) should be suitable for such research.

## 2. Materials and Methods

In selecting the materials to be tested, there was an effort to take into account their different deformation behaviour and also their different microstructures. For this reason, representatives of high-strength and deep-drawing materials, commonly used in car body design, were selected. Specifically, two-phase steel CR290Y490T with a martensitic-ferritic structure and a thickness of 0.55 mm, later referred to as DP500, was tested. As a deep-drawing material, the ferritic steel CR4EG29-29-E-P-O with a thickness of 0.70 mm, further referred to as CR4, was then chosen. The actual carried out experiments and the methods used to evaluate them are described in the following chapters.

### 2.1. Static Tensile Test

**Figure 1:**
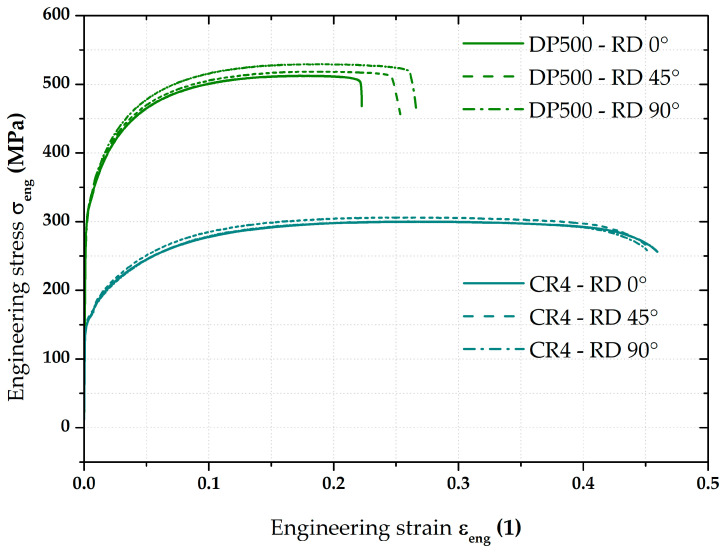
Engineering stress–strain curves from the static tensile test for tested material.

At first, the static tensile test was used to determine the basic material properties of the tested materials. Taking into account the anisotropic behaviour of both materials, three basic directions relative to the rolling direction (RD) were used in the test—0°, 45°, and 90°. Five specimens were tested for each measured rolling direction. The actual tensile tests were carried out in accordance with EN ISO 6891-1 [45] on a modernised testing device TIRA Test 2300 (Schalkau, Germany) using software Labtest v.4 (LabControl, Opava, Czech Republic) for evaluation, which allows the required basic material properties (proof yield strength *σ*_p0.2_, ultimate tensile strength *σ*_m_, total ductility *A*_80mm_, uniform ductility *A*_g_, and Young’s modulus of elasticity *E*) to be accurately determined. The required measured values are summarised in Table 1. Engineering stress–strain curves from the static tensile test of both tested materials are plotted in Figure 1. In this case, averaged curves from 5 measurements are shown.

Concerning the main focus of this paper, which is to determine the effect of loading mode on the position of the stress–strain curve at HBT (i.e., under equi-biaxial stress state), the true stress–strain curves determined by the static tensile test (uniaxial tensile stress state) were not important. However, these data have been also processed as part of the planned further research, where one of the areas to be investigated is the accurate definition of the yield conditions.

**Table 1:** Basic material properties of the tested materials DP500 and CR4.

Material	RD (°)	*σ*_p0.2_ (MPa)	*σ*_m_ (MPa)	*A*_g_ (%)	*A*_80mm_ (%)	*E* (MPa)
**DP500**	0	318.5	514.6	17.79	22.97	199,180
	45	319.6	521.3	18.62	25.55	212,450
	90	314.2	528.9	18.59	26.15	221,569
**CR4**	0	157.7	301.1	26.76	46.12	183,920
	45	156.7	306.7	26.06	45.46	205,239
	90	154.6	300.6	26.16	46.55	191,942

### 2.2. Hydraulic Bulge Test (HBT)

The values from the static tensile test represent the basic material properties. However, the focus of this research was to determine biaxial stress–strain curves. For this reason, all further experiments were carried out using the hydraulic bulge test (HBT), in which the specimen is subjected to an equi-biaxial stress state. The initial workpiece for the tests is a rounded blank with a diameter of 210 mm, made by shearing. Since the optical system GOM Correlate Pro (Carl Zeiss GOM Metrology GmbH, Germany) was used for strain analysis, it was necessary to first degrease the specimens and then apply a stochastic pattern. For the actual test and subsequent evaluation, rolling direction (RD) was also marked on each specimen before testing. During the test, the values of pressure and deformation induced on the specimen surface were recorded synchronously by cameras (DIC—Digital Image Correlation). From these input data, the required stress–strain curves can be determined and calculated. The following equations were used.

**Figure 2:**
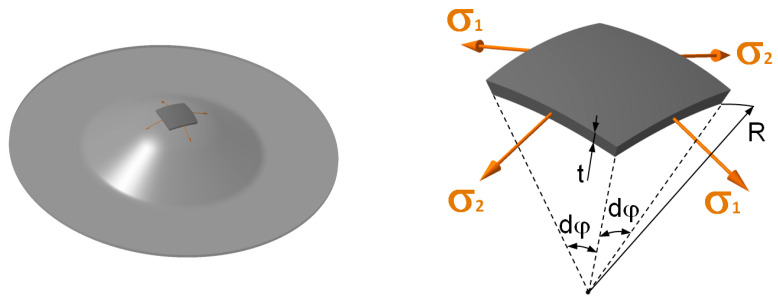
Principle and stress state at the hydraulic bulge test (HBT).

Following Figure 2, which shows the effect of fluid pressure on the stress distribution for the selected element, Equation (1) can then be derived using the equilibrium equation to calculate the biaxial true stress *σ_BT_* in the specimen wall.

(1) $$\sigma_{BT}=\frac{p\cdot R}{2\cdot t}$$

where:

*p*—hydraulic pressure (MPa);

*R*—radius of curvature (mm);

*t*—actual thickness of specimen (mm).

Values of major and minor true strains *ε*_1_ and *ε*_2_ are determined by means of contactless optical system. Using the law of volume constancy and neglecting the elastic deformation, true thickness strain *ε*_3_ can be calculated according to Equation (2):

(2) $$\varepsilon_{3}\approx -\left(\varepsilon_{1}+\varepsilon_{2}\right)$$

In terms of deformation, the plastic work principle is used for the resulting biaxial stress–strain curves according to standard ČSN EN ISO 16808 to express the (true) plastic strain *ε_pl_*—see Equation (3). Final biaxial stress–strain curves were plotted as *σ_BT_* vs. −(*ε_pl_*).

(3) $$\varepsilon_{pl}=-\left(\varepsilon_{1}+\varepsilon_{2}\right)+2\frac{1-\nu }{E}\cdot \sigma_{BT}$$

where:

*ν*—Poisson’s ratio (1)

*E*—Young’s modulus (MPa)

**Figure 3:**
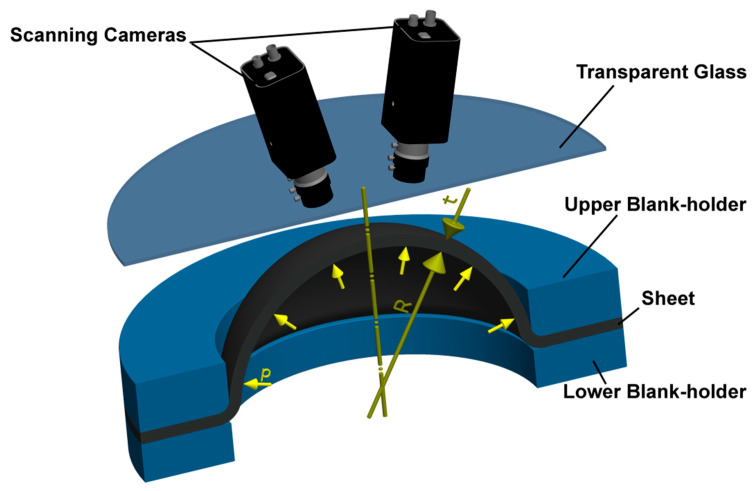
Scheme of the measurement of the biaxial stress–strain curve at HBT via DIC.

As was already written above, to determine the biaxial stress–strain curves during the hydraulic bulge test, a contactless optical system for measurement deformation was used—in this case, GOM Correlate Pro from German company ZEISS GOM. The schematic layout of the actual test is shown in Figure 3.

During the measurement of deformation characteristics, the given jig was placed on the bolster of hydraulic press CBA 300/63. The magnitude of the applied clamping force was 2000 kN, which is, for the tested materials, high enough to prevent the specimen from sliding out of the clamping area. The diameter of the die was 100 mm and, thus, the surface radius values were as follows: r_1_ = 12.5 mm (for determining curvature) and r_2_ = 5 mm (for determining strain). It is necessary to vent the whole system before every measurement. As external equipment for this press, there was a programmable and own press-independent hydraulic unit that controlled the pressure courses in the jig. A precision Proportional Integral Derivative (PID)-controlled hydraulic servo valve with an achievable accuracy of pressure 0.002 MPa ensured the accurate adjustment and progression during the pressure loading of the specimen. The actual deformation process was scanned by a pair of synchronised 12 MPx resolution digital cameras with 20 mm lenses. These cameras were positioned at the required distance from the specimen to be measured (450 mm) using aluminium profiles. The distance between the cameras was 220 mm. Since the optical system operates with a fixed focal length, it is always necessary to accurately adjust not only the lighting conditions but, also, for example, the aperture size in the given measuring volume. Last but not least, it is important to focus the optical system carefully on the surface of the test specimen. Before performing the HBT itself, it is then always necessary to calibrate the used optics, which, in this case, was conducted using a relevant calibration plate (CP40/170/44142), whose position in space was scanned by cameras during calibration. Depending mainly on the used calibration plate point spacing, the distance of cameras from the object to be measured and from each other, a so-called calibration volume was then obtained, which, in this case, was as follows: 160 mm × 120 mm × 100 mm (length × width × depth). The system also automatically evaluated the calibration quality. The measurement error according to the calibration protocol was, in this case, 0.049 Px. When measuring the biaxial stress–strain curves (generally when external data also need to be processed), it is necessary to have the actual value of applied pressure for each pair of images during the test.

**Figure 4:**
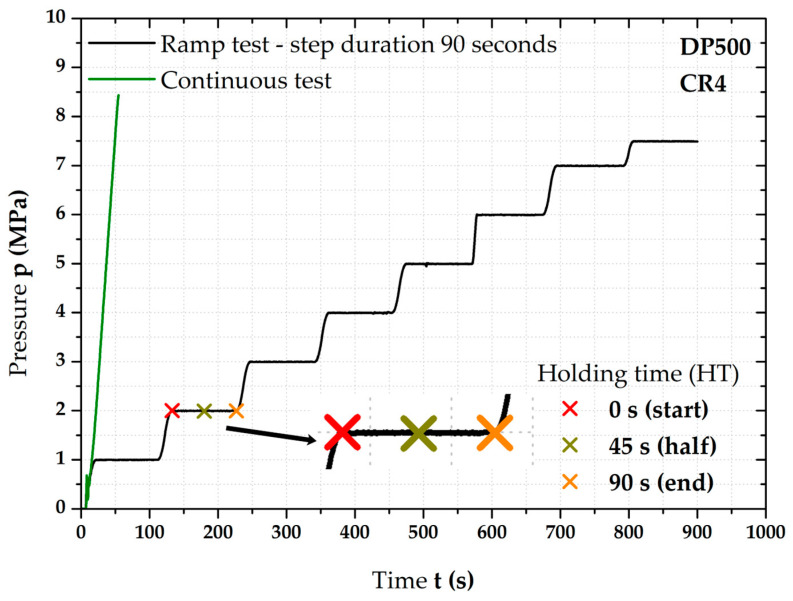
Comparison of used loading modes—continuous measurement and ramp test.

The major focus of this paper was to find the influence of loading mode on the position of the biaxial stress–strain curve. Because of this, these curves were measured under two different loading modes, which are shown in Figure 4 and were as follows:

- **Continuous tests**—“classical” type of loading that was carried out according to ISO 16808;
- **Ramp tests**—at every pressure step, there was always applied a holding time of 90 s.

In light of ramp tests, it has subsequently proved appropriate to divide these tests into several subgroups, which were as follows:

- **Ramp test without HT (holding time)**—without the influence of applied holding time;
- **Ramp tests with HT** —three important time moments were taken into account
                —**start** of HT (**0 s**); **half** of HT (**45 s**); and **end** of HT (**90 s**).

So, during so-called continuous tests, a constant increase in pressure depending on time was used. For both tested materials (DP500 and CR4), these kinds of tests were always carried out up to fracture because of Forming Limit Diagrams (FLC).

However, besides the common continuous tests, so-called ramp tests were investigated as well. In this case, it meant that, on every integer multiple of pressure value (from 1 MPa up to 7 MPa), a holding time (HT) was used—step duration of 90 s. The last applied pressure was 7.5 MPa. In the case of the ramp test, a pressure of 8 MPa was not used because there was a risk of specimen failure during the holding time. Subsequently, all determined stress–strain curves were fitted by the Hollomon equation—see Equation (4)—up to a pressure value of 7.5 MPa, although the actual failure of the material under continuous measurement occurred at approximately 8.5 MPa (see Figure 4).

(4) $$\sigma_{BT}=K\cdot \varepsilon_{pl}^{n}$$

where:

K—strength coefficient (MPa);

*n*—strain hardening exponent (1).

From Figure 4, it is also evident that the ramp test itself was very time-consuming; in this case, one test lasted up to about 900 s. Given the resulting size of the measurement (due to the large number of acquired frames), it was suitable to set the frame rate to 3 fps. An example of observed time moments for 2 MPa is also shown in Figure 4.

As can be seen, the selected loading modes were quite very different in terms of time course. For these reasons, it was clear that the selection of loading method would result in different strain rate evolutions, with a gradual increase in the case of continuous measurement (slowly at first to relatively high values just before the failure) and then only certain “peaks” at moments of increasing pressure values during the ramp tests. The actual strain rate courses for two applied loading methods are shown below. In addition to that, ramp tests themselves were very time-consuming. Because of that, 5 continuous as well as 5 ramp tests for both tested materials were carried out and resulting stress–strain curves were taken as average curves from these measurements.

## 3. Results

In this chapter, the major results measured for the tested materials and via the testing methods mentioned above are described. These final results can be classified as follows:

●
**Strain rates**
- values of strain rates resulting from the used loading modes;●**∆R** and **∆Z**- the change in radius of curvature and maximal dZ position (thus, height of dome) gave a first basic overview of the deformation behaviour of tested materials;●
**Stress-strain curves**
- the main monitored results were determined under the different loading modes●
**SEM analysis**
- evaluation of microstructure to better describe the final deformation behaviour of tested materials in light of microstructure (grain size, orientation of grains, etc.)- an area of 250 × 250 µm^2^ was selected for scanning and the step size was 0.2 µm.

### 3.1. Courses of Strain Rates Depending on the Loading Mode

**Figure 5:**
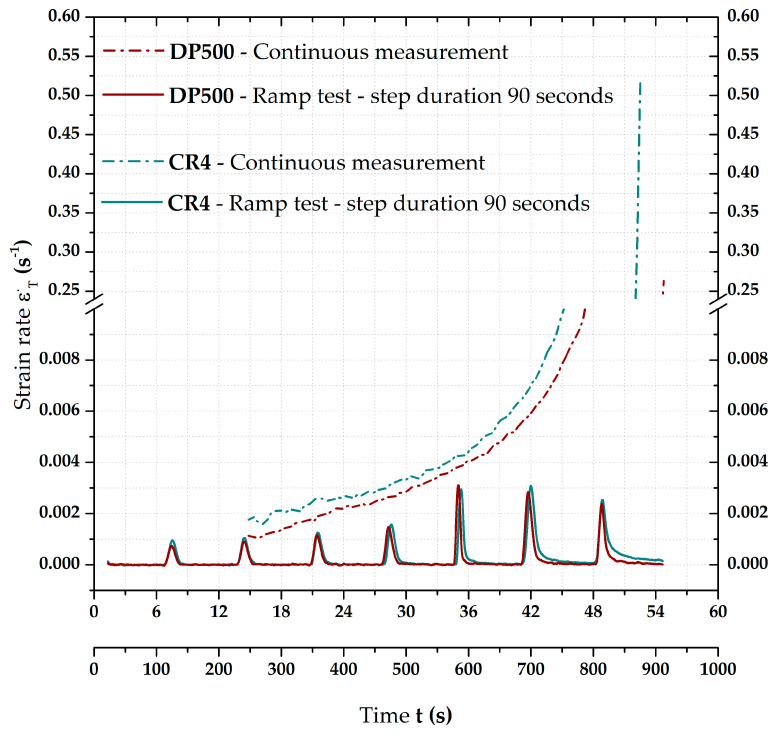
Comparison of strain rates for continuous measurement and ramp test.

As was already mentioned, the first important characteristics were the values of strain rates, which resulted from the applied loading modes. Figure 5 shows graphically the strain rates resulting from the used loading methods, i.e., for the continuous measurement and ramp test. Due to the relatively different values of time and strain rates for these two approaches, it was first necessary to use two X-axes (for time) in this graph, the upper one for the continuous measurement and the lower one for the ramp test. It was also necessary to break the *Y*-axis to show low as well as high strain rate values. Both curves are plotted just after reaching a pressure of 1 MPa because there was venting of the whole system before that. It can be seen that, at the beginning, there was a gradual increase in strain rate during the continuous measurement, which, however, revealed an almost exponential increase at the end of test, reaching 0.515 s^−1^ for CR4 and 0.263 s^−1^ for DP500 just before the crack. Compared to the continuous measurement, the ramp tests at first sight show changes in light of strain rate only during the pressure increase. However, already, in Figure 5, it is evident that, as the pressure increases, the strain rate values also gradually increase—already during the holding time.

**Figure 6:**
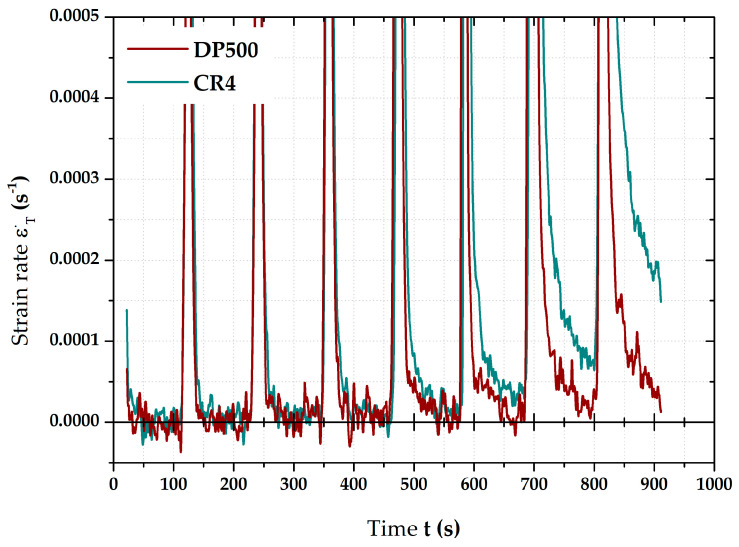
Detailed comparison of strain rates during the holding times.

That is why Figure 6 shows strain rate courses only of ramp tests for both tested materials in great detail. The position of strain rate zero values is also highlighted there. Almost the same trend in the deformation behaviour of both materials is quite evident, i.e., the higher the pressure values for holding times, the higher the mean strain rates during these time periods. During the first pressures (cca up to 4 MPa), it can be observed that strain rate values return to zero values before the end of relevant HT. However, this is not valid for the remaining pressure values. There are still higher and higher magnitudes of strain rates for both materials during HT. Nevertheless, little different deformation behaviours of the tested material can be observed. First of all is just about the own strain rate values, where deep-drawing material CR4 reveals quite higher values than high-strength steel DP500. The higher the pressure, the bigger the difference between them. In addition to that, there is one more difference regarding the ability of the material to reduce the strain rate to zero during HT. It seems that DP500 can reduce these values to zero already under high pressure values. In contrast, this is not true in the case of CR4, where strain rate magnitude is still quite significant at the end of relevant HT.

### 3.2. Material Behaviour During the Step Duration at Ramp Test

**Figure 7:**
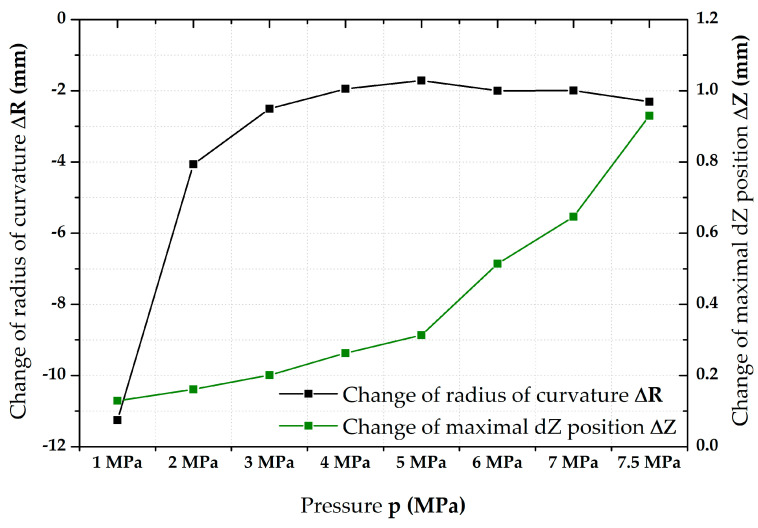
DP500—change in R (mm) and dZ (mm) during step duration.

**Figure 8:**
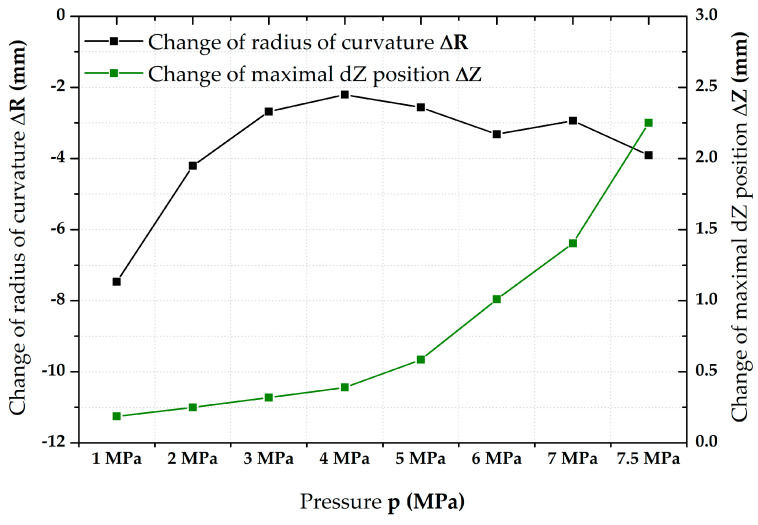
CR4—change in R (mm) and dZ (mm) during step duration.

As has been already written above, the major results of this paper are about the description of the biaxial stress–strain curves—see Section 3.3 and Section 3.4. Nevertheless, the material behaviour during the step duration at the ramp test was also monitored. Because of this, the change in radius of curvature R (mm) as well as the change in maximal dZ position (mm) depending on time were monitored as well. Since the 3-2-1 registration of each specimen used as the basic plane the initial surface of a specimen, this was actually also the current height of the dome. The radius of curvature R was determined right from the contactless deformation measurement via the construction best-fit sphere in the desired area (surface radius r_1_). In contrast, the maximal dZ position represents just the maximal value in the *Z*-axis (height of the dome). Differences were always taken between these values at the moments 0 s (start) and 90 s (end) of holding time (HT) on the relevant pressure values. Examples of the final values for the first samples of both tested materials are given in Table 2 and Table 3. Initial (x_0_), final (x_1_), and difference (dx) values are always shown. These results are shown graphically in Figure 7 (DP500) and Figure 8 (CR4).

**Figure 9:**
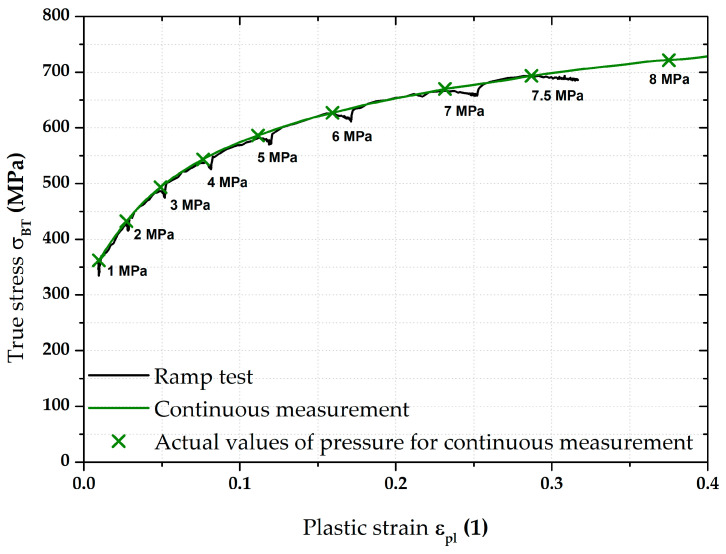
DP500—overview of the major results.

The initial results described above regarding the change in hemispherical geometry of the specimen during HT at a given pressure are summarised in the following pages. Table 2 shows the values for the change in the radius of curvature R and the maximum change in *Z*-axis (thus, height of the dome) for the DP500 material. For the radius of curvature values, it can be seen that the largest changes during the step duration (90 s) occur during the first three applied pressures (up to about 3 MPa) and then there is some stabilisation of this change at about 2 mm. In the case of biaxial stress–strain curves, the EN ISO 16808 standard was already followed and, thus, two surface radii were used (to be specific, r_1_ = 12.5 mm and r_2_ = 5 mm). Since the magnitude of σ_BT_ during HBT is dependent on the radius of curvature—see Equation (1)—it decreases during the holding time. This is particularly significant at lower pressures where the change in radius of curvature is greater. For the change in the maximum *Z*-axis position (thus, height of the dome), this trend is reversed with increasing increments as the pressure increases. Therefore, when the biaxial stress–strain curve was measured, the curve during HT revealed change mostly only in the strain direction (*X*-axis)—see Figure 9.

**Table 2:** DP500—radius of curvature and maximal dZ position—change during HT.

Applied Pressure	Radius of Curvature R (mm)			Maximal dZ Position dZ (mm)		
p (MPa)	R_0_	R_1_	ΔR	dZ_0_	dZ_1_	ΔZ
**1**	387.071	375.815	**−11.256**	4.442	4.570	**0.129**
**2**	230.140	226.074	**−4.067**	7.723	7.884	**0.161**
**3**	170.633	168.125	**−2.508**	10.565	10.767	**0.201**
**4**	136.962	135.011	**−1.951**	13.306	13.569	**0.263**
**5**	114.380	112.666	**−1.713**	16.141	16.454	**0.313**
**6**	97.643	95.643	**−2.000**	19.057	19.571	**0.514**
**7**	82.649	80.651	**−1.997**	22.793	23.439	**0.646**
**7.5**	75.750	73.438	**−2.312**	25.052	25.981	**0.930**

The values of change in radius of curvature R and maximum *Z*-axis position (height of dome) for the deep-drawing material CR4 are given in Table 3. In terms of these observed quantities, this material shows similar behaviour to the high-strength material DP500. The differences are almost only in the absolute values of these monitored quantities. However, the change in the radius of curvature R for the other pressures does not show such stabilisation as in the case of DP500 after an initial sharp decrease up to 4 MPa and a gradual increase in the values of these increments can be observed (except for 7 MPa) up to a final increase of 3.9 mm in the case of 7.5 MPa. The values of change in the maximum *Z*-axis position for the material CR4 again show the same trend as in the previous case. However, it can be seen that the absolute magnitudes of these increments are considerably higher than in the previous case. Again, the higher the pressure value, the greater the change in the maximum *Z*-axis position (height of the dome) of the material. In terms of the biaxial stress–strain curve’s resulting shape, it can again be seen that the effect of the change in radius of curvature (reduction in stress values) is predominant at lower pressures. Conversely, at higher pressures (around 5 MPa and above), the effect of changing the height of the dome is more significant, resulting again in elongation of the biaxial stress–strain curve in the direction of higher strain during HT.

**Table 3:** CR4—radius of curvature and maximal dZ position—change during HT.

Applied Pressure	Radius of Curvature R (mm)			Maximal dZ Position dZ (mm)		
p (MPa)	R_0_	R_1_	ΔR	dZ_0_	dZ_1_	ΔZ
**1**	289.625	282.154	**−7.471**	5.848	6.034	**0.186**
**2**	183.485	179.279	**−4.206**	9.767	10.016	**0.249**
**3**	140.298	137.619	**−2.680**	13.041	13.358	**0.318**
**4**	115.096	112.889	**−2.206**	16.152	16.540	**0.389**
**5**	97.690	95.126	**−2.564**	19.283	19.868	**0.584**
**6**	84.779	81.459	**−3.319**	22.565	23.574	**1.009**
**7**	72.135	69.197	**−2.938**	27.155	28.559	**1.404**
**7.5**	65.930	62.022	**−3.908**	30.326	32.576	**2.250**

### 3.3. Stress–Strain Curves—Results for DP500

The most important results were about the final position of equi-biaxial stress–strain curves and such results are summarised on the following pages. For clarity, Figure 9 shows the two main methods used to measure the stress–strain curves. The flow curve measured from the continuous measurement was always used as the reference for all measurements. This “classical” approach (in accordance with EN ISO 16808) is shown in olive green for all graphs. For a better overview, the moments where the monitored pressure values were reached during the test are shown on this stress–strain curve as well. The second used loading mode (referred to as the ramp test) then monitored the effect of changing loading course, where, instead of a classic continuous increase in pressure, a holding time for 90 s was applied right again in the moments when the monitored pressure was reached (see, e.g., Figure 9). The evaluation always started at the first applied pressure (1 MPa). The last monitored pressure in this case was 7.5 MPa. For this reason, the stress–strain curves from the continuous measurements are shown only up to the value ε_pl_ = 0.4, although these curves were already measured up to the moment of fracture, as it was assumed that these data would be also used in the Forming Limit Diagrams (FLDs). Five samples for both applied loading modes were always measured in this way and a basic comparison of their average stress–strain curves is shown in Figure 9.

**Figure 10:**
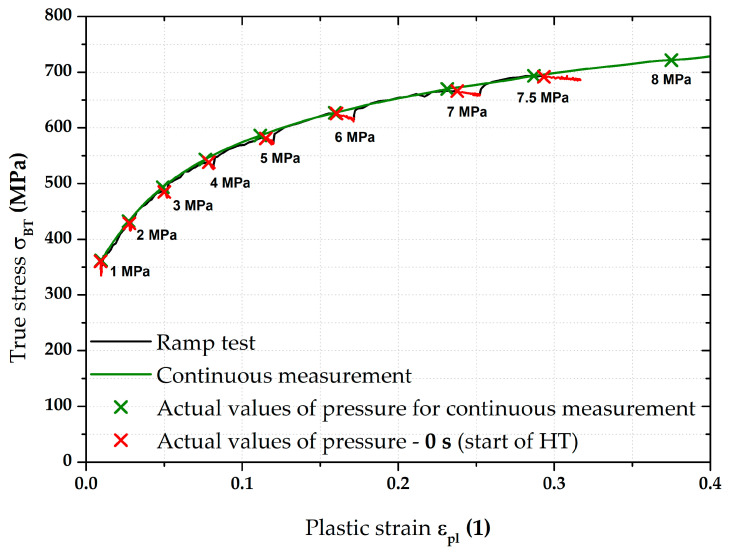
DP500—continuous measurement vs. ramp test with holding times.

Figure 9 above clearly shows the deformation behaviour of material DP500 over the HT applied during ramp tests. A more detailed graphical representation of these holding times at constant pressure is shown in Figure 10, where the stress–strain curve from the ramp test is compared with the continuous measurement. In addition to the previously described quantities, the start of each holding time within the ramp test is also shown (red crosses for the relevant pressures) and the actual holding time at a given pressure is graphically highlighted in red. From this point of view, it is particularly interesting to monitor the change in the effect of holding time (for each 90 s pressure) with respect to the position of a given stress–strain curve area. At lower pressure values, this effect is more likely to be seen as a decrease in the stress region (direction of the −*Y*-axis), while, at higher pressure values, the change is more likely to be seen as strain (direction of the +*X*-axis).

**Figure 11:**
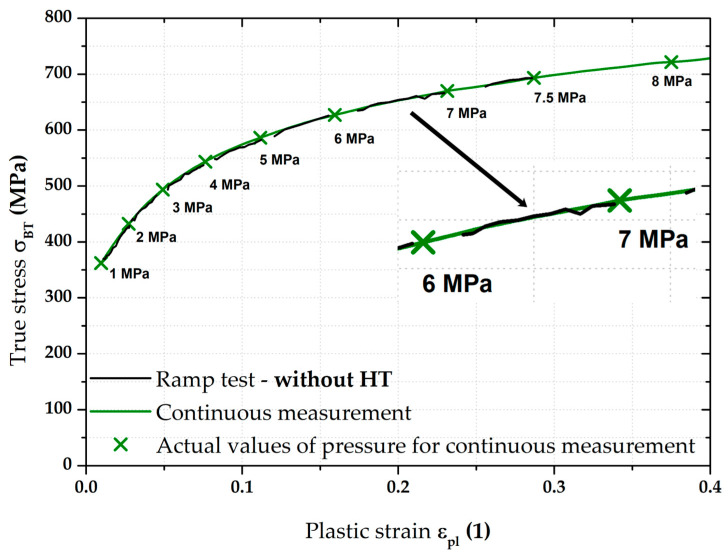
DP500—continuous measurement vs. ramp test without holding time.

In light of the material deformation behaviour, it is interesting to take into account the position of the stress–strain curve without the influence of applied holding time. It is shown in Figure 11, which also gives detail of the area between 6 and 7 MPa. It can be seen that these “crop” values of the ramp test almost exactly match the stress–strain curve from the continuous measurement. This fact is then mathematically expressed with the help of fitting; Hollomon equation was used in this paper.

**Figure 12:**
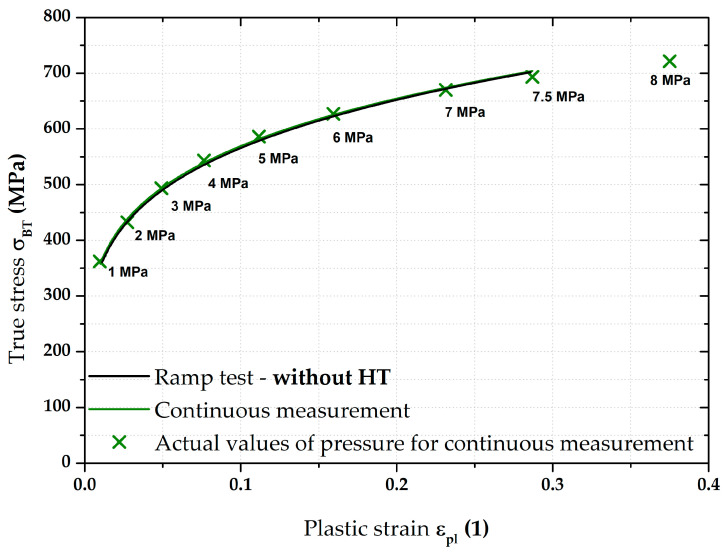
DP500—continuous measurement vs. ramp test without HT (fitting curves).

Figure 12 then compares the continuous measurement and the ramp test but now only up to a pressure of 7.5 MPa and the Hollomon equation is used to approximate the area of plastic strain. These two curves overlap almost exactly. The actual numerical values of strength coefficient C and strain hardening exponent n are given in Table 4.

**Figure 13:**
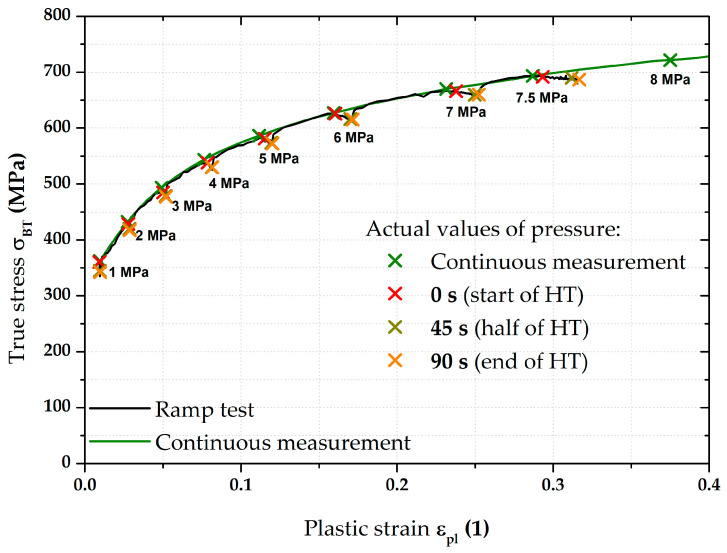
DP500—overview of the monitored time moments during holding time.

The next step was then to fit the stress–strain curves according to the Hollomon equation right during the holding times (thus, using the constant pressure values). For the purposes of this paper, time moments of 0 s (start), 45 s (half), and after 90 s (end) of these holding times were selected. These time moments are plotted graphically in Figure 13.

Although there were not many points for fitting these stress–strain curves, the main point in this case was to show the actual trend in light of the holding time’s effect. The computed values of the fitting constants (C and n) also for these three other monitored time moments during the holding time are shown in Table 4.

**Table 4:** DP500—values of fitting constants (C, n) for all monitored loading modes.

Measuring Mode	Continuous Measurement	Ramp Test—Without HT	0 s (Start of HT)	45 s (Half of HT)	90 s (End of HT)
**C (MPa)**	904.5	907.4	884.3	876.6	874.6
**n (1)**	0.2013	0.2053	0.1963	0.2025	0.2036

**Figure 14:**
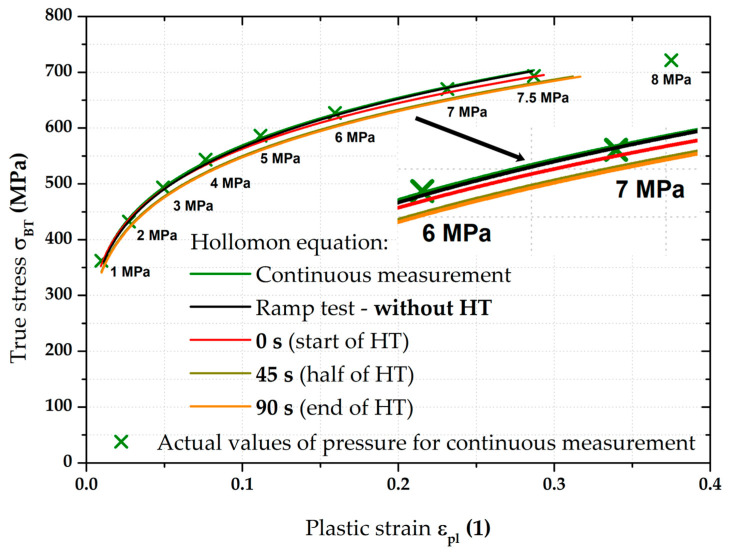
DP500—fitting curves for all monitored loading modes.

A graphical summary of all determined stress–strain curves can be seen in Figure 14. In addition to the continuous measurement and ramp test without the influence of used holding times (already described above), stress–strain curves are also shown for the chosen time moments of HT (colours of these curves correspond to the colours used for crosses in Figure 13). For better illustration, the detail of the range between 6 and 7 MPa is also used in this case. It is quite interesting that the effect of the first 45 s of HT at a given pressure is much more pronounced than the change in the stress–strain curve during the rest of HT (46–90 s). It can be seen that the curves for the half and end of holding time are almost indistinguishable, which is numerically confirmed by the strength coefficient values for these loading modes (see Table 4). This fact can already be observed in Figure 13, where the very close position of crosses for the half and end of the relevant holding times can be seen. From these results, it can be concluded that, within the holding times (90 s in this case), a significant part of the change in radius of curvature R (or position in the *Z*-axis direction—height of the dome) occurs within the first 45 s. In the context of these results, it would be suitable to focus future attention also on the dynamics of this effect, as this issue was not further discussed in this paper (regarding its reasonable length).

### 3.4. Stress–Strain Curves—Results for CR4

**Figure 15:**
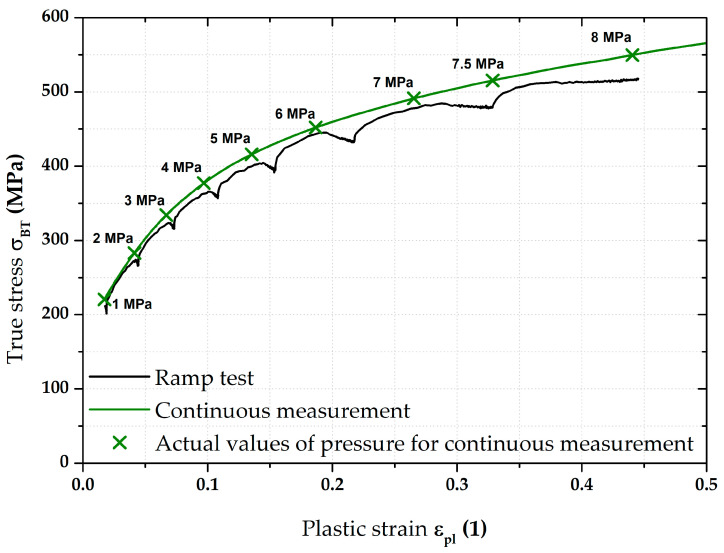
CR4—overview of the major results.

The main stress–strain curves obtained for the deep-drawing material CR4 are shown in Figure 15. As a reference stress–strain curve, the result of the continuous measurement is again taken (shown in olive green). Against this reference, the result of the average stress–strain curve from the ramp test is shown in the same colour as in the case of DP500. In this case, the change in the positions of stress–strain curves from the ramp test can be seen at a glance. Holding times of 90 s were used again for the same pressure values as in the previous case, which are also shown in the following graphs. It is obvious that, in this case, there was no return of deformation behaviour to the stress–strain curve obtained from the continuous measurement after the holding time. As the ramp tests ended also after 90 s at 7.5 MPa, the plastic strain ε_pl_ values in this case are limited to 0.5. The specimens used for the continuous measurements were also tested up to failure and these data were subsequently used in creating FLDs.

**Figure 16:**
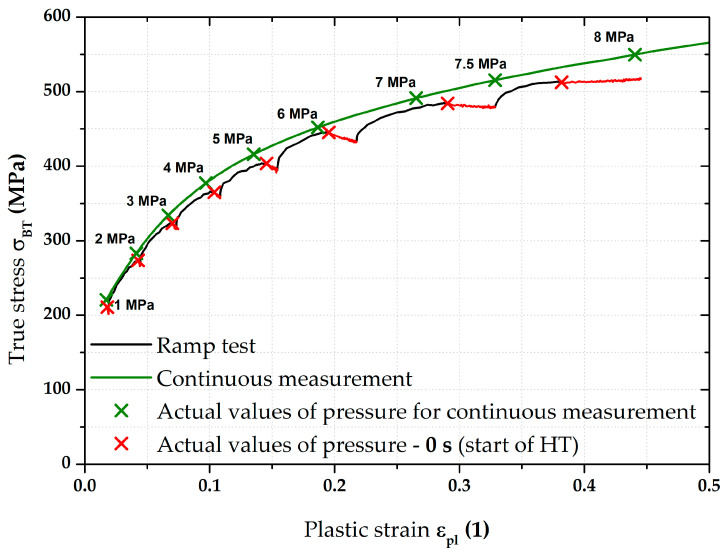
CR4—continuous measurement vs. ramp test with holding times.

Figure 16 then shows, in higher detail, the biaxial stress–strain curve from the ramp test. The positions of red crosses indicate again 0 s (start), 45 s (half), and 90 s (end) for holding times at each pressure value. Comparing the corresponding pairs of selected points, a fairly significant shift in the position of the stress–strain curve due to the used loading mode can be seen in this case.

For a better overview, Figure 16 also graphically shows the actual holding times at each applied pressure in red. In general, the trend is the same as that observed for the material DP500 (see Figure 10), i.e., a decrease mainly in stress (*Y*-axis) at lower pressures and then a change mainly in strain (+*X*-axis) at higher pressures. On the other hand, it can be seen that these strain changes are greater in the case of deep-drawing material CR4.

**Figure 17:**
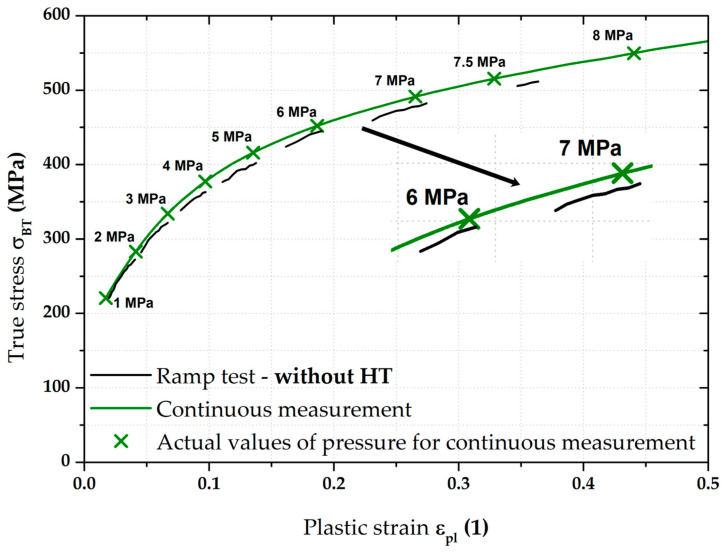
CR4—continuous measurement vs. ramp test without holding time.

Of course, the effect of the loading mode for material CR4 was again monitored by determining the resulting position of the biaxial stress–strain curve itself measured by the ramp test but, again, without taking into account the effect of HT. This result is shown graphically in Figure 17 as part of its comparison with the continuous measurement.

**Figure 18:**
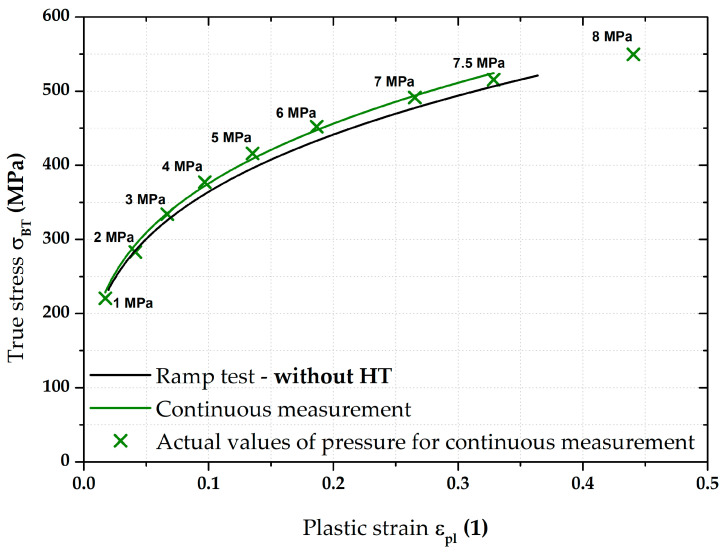
CR4—continuous measurement vs. ramp test without HT (fitting curves).

From Figure 17, it is evident that, for the deep-drawing material CR4, the loading method has a fairly significant effect on the difference between the continuous measurement and the ramp test. Approximations of these two stress–strain curves using the Hollomon equation are shown in Figure 18. The values of fitting constants are then given in Table 5.

**Figure 19:**
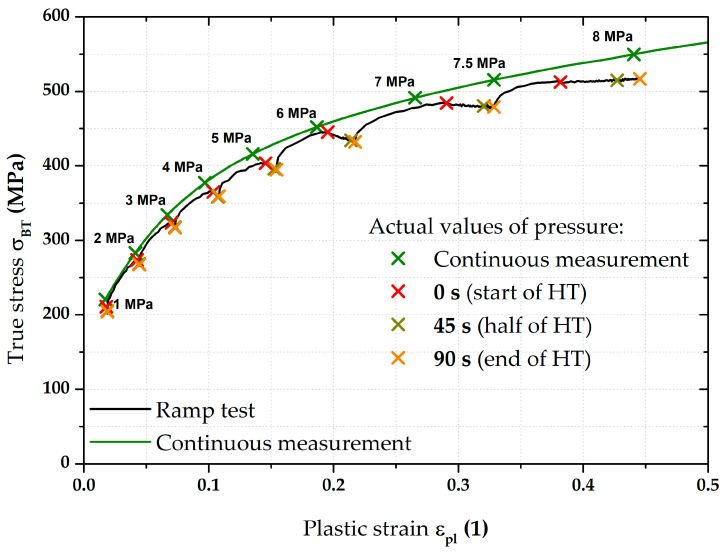
CR4—overview of the monitored pressure values during HT.

Moreover, the influence of HT was observed as well, again using the same time moments—0 s (start), 45 s (half), and 90 s (end) of HT. These observed moments are then plotted in Figure 19. Once more, there can be seen not only a change in slope at HT but, also, certain approaches of half and end moments regarding the deformation behaviour.

Using the given moments of the start, half, and end of the holding times, it was again possible to determine approximations of the stress–strain curves using the Hollomon equations of these states. All the final values of fitting constants are then given in Table 5, together with the results of the continuous measurements and the ramp test without the effect of used holding times.

**Table 5:** CR4—values of fitting constants (C, n) for all monitored loading modes.

Measuring Mode	Continuous Measurement	Ramp Test—Without HT	0 s (Start of HT)	45 s (Half of HT)	90 s (End of HT)
**C (MPa)**	716.8	689.5	690.9	667.0	659.8
**n (1)**	0.2809	0.2772	0.2866	0.2862	0.2838

**Figure 20:**
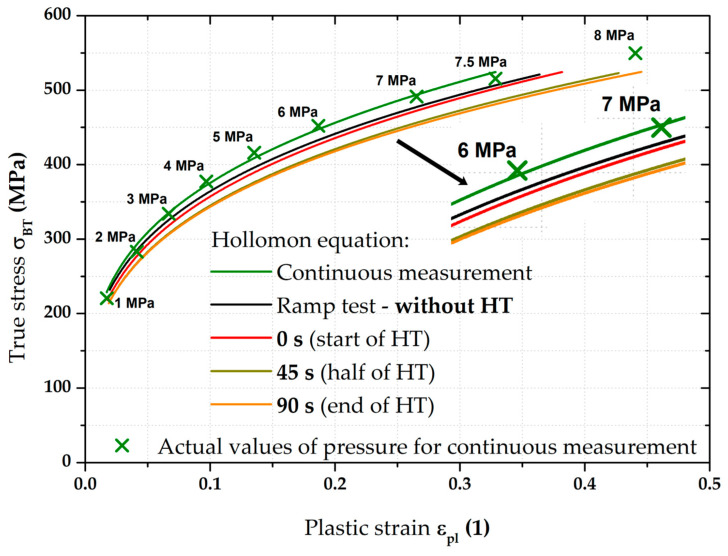
CR4—fitting curves for all monitored loading modes.

A comparison of all monitored approximations of stress–strain curves for CR4 using the Hollomon equation is shown in Figure 20. There is obviously a gradual decrease within these curves due to the decreasing value of strength coefficient as well as the different deformation behaviour between continuous measurement and ramp tests without the influence of HT. In contrast, the results for half and end of HT almost overlap again.

### 3.5. Evaluation of Microstructure

The microstructure was investigated using a scanning electron microscope (SEM) TESCAN MIRA 3. Not only were specimens compared before and after deformation for both tested materials (DP500 and CR4) but also specimens with different loading modes, i.e., continuous measurement and ramp test. An area of 250 × 250 µm^2^ was selected for scanning and the step size was 0.2 µm.

**Figure 21:**
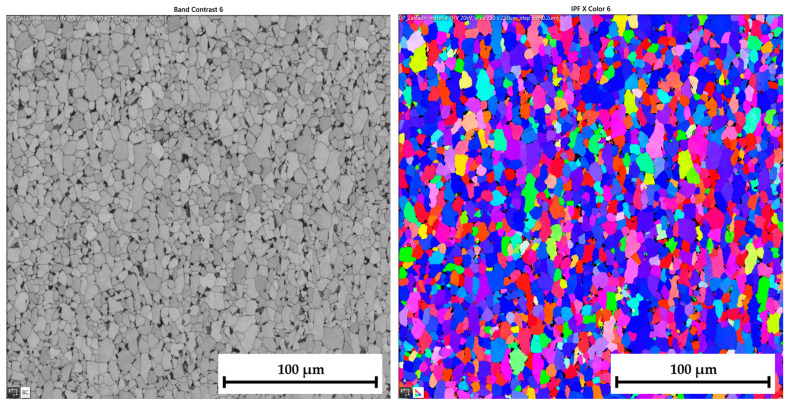
**DP500 before deformation**—microstructure of basic material (**left**) and IPF analysis of grain orientation in the **X-direction** (**right**).

**Figure 22:**
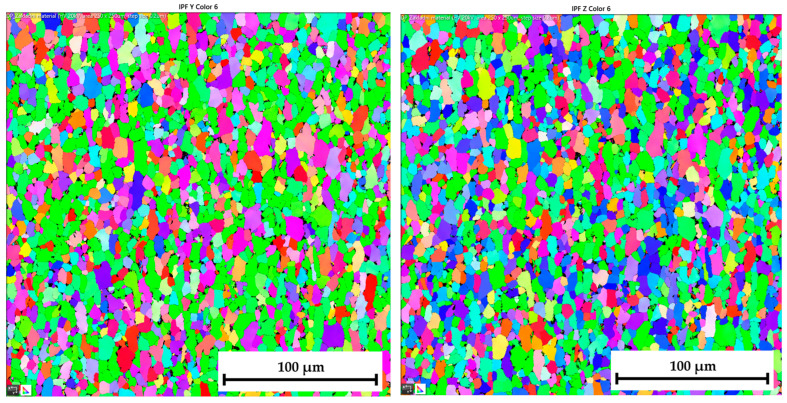
**DP500 before deformation**—IPF analysis of grain orientation in the **Y-direction** (**left**) and in the Z-direction (**right**).

The ferritic-martensitic microstructure (with a ferrite content of 95.5%) of material DP500 before deformation is shown by Electron Backscatter Diffraction (EBSD) analysis in Figure 21 (left). In addition to EBSD analysis, Inverse Pole Figure (IPF) analysis was also performed to show the grain orientation in the material. The orientation of the grains can be seen in Figure 21 (right) for X-direction. Such an orientation in the Y-direction and Z-direction is shown in Figure 22. It can be seen that this is a typical orientation of the material produced by the rolling technology, where the sheet thickness direction is given in the X-direction and RD 0° is in the Y-direction. Images from IPF analysis for grain orientations in the Z-direction (thus, perpendicular to RD 0°) are shown here also but only for the basic materials because they were not so important for the research. Generally, typical colour indication of grain orientation was used according to the RGB approach; thus, red colour corresponds to (001), green is for (011), and, finally, blue is for (111).

**Figure 23:**
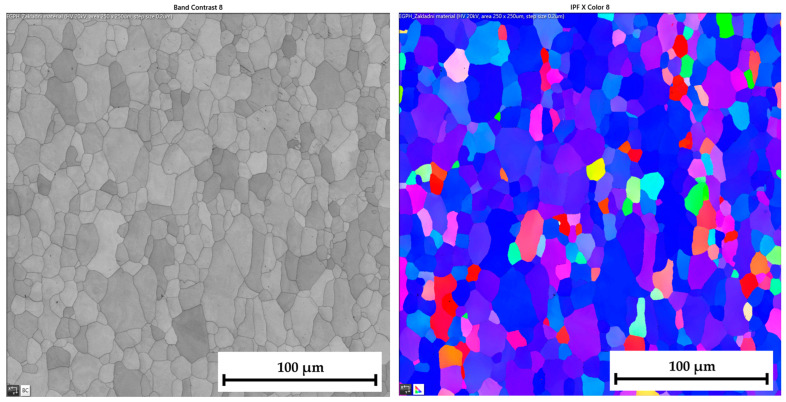
**CR4 before deformation**—microstructure of basic material (**left**) and IPF analysis of grain orientation in the **X-direction** (**right**).

Similar to the material DP500, the initial microstructure of deep-drawing material CR4 was analysed as well. The microstructure of CR4 is formed almost by a pure ferritic structure (99.7%), which can be seen in Figure 23 (left).

**Figure 24:**
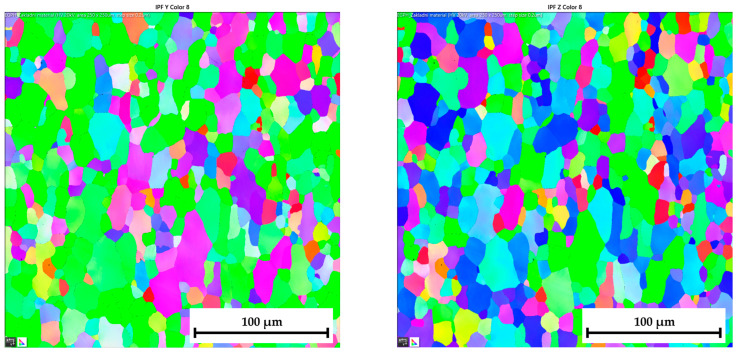
**CR4 before deformation**—IPF analysis of grain orientation in the **Y-direction** (**left**) and in the **Z-direction** (**right**).

The IPF analysis to reveal the grain orientation in the deep-drawing material CR4 is shown in Figure 23 (right) for X-direction and in Figure 24 both for Y-direction (left) and Z-direction (right). Here, it can be seen that this is again the typical orientation of the material produced by rolling technology, where the sheet thickness is given in the X-direction and RD is again in the Y-direction. The initial grain size values are shown in Table 7. From the measured values, the result is that the initial grain size dimensions (breadth × length) of material CR4 are approximately two times higher than those of the material DP500.

**Figure 25:**
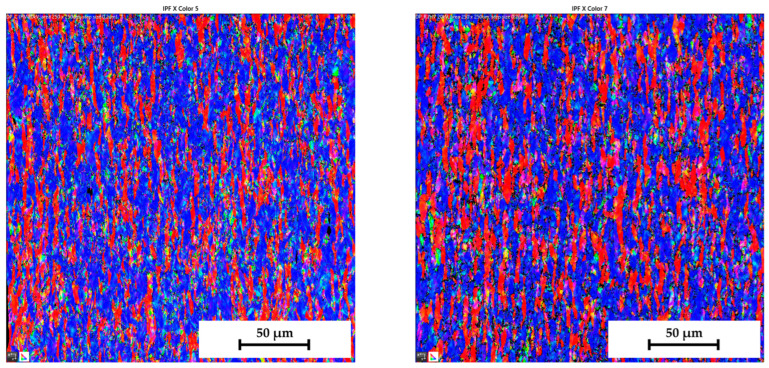
DP500 after deformation—IPF analysis of grain orientation in the X-direction (**left**—continuous measurement, **right**—ramp test).

**Figure 26:**
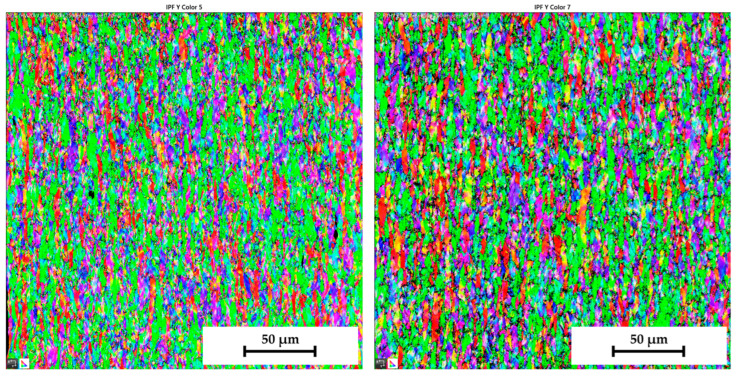
DP500 after deformation—IPF analysis of grain orientation in the Y-direction (**left**—continuous measurement, **right**—ramp test).

**Figure 27:**
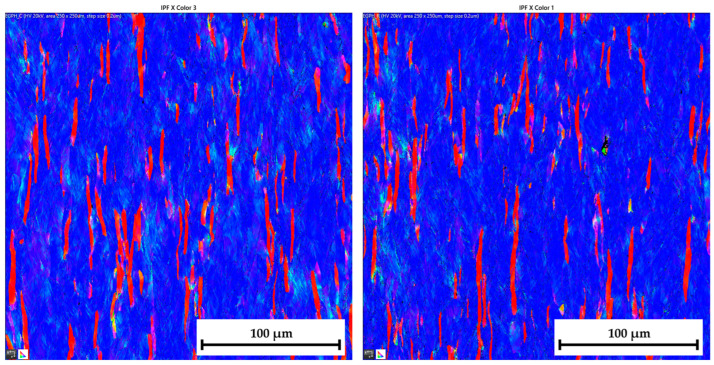
**CR4 after deformation**—IPF analysis of grain orientation in the **X-direction** (**left**—continuous measurement, **right**—ramp test).

**Figure 28:**
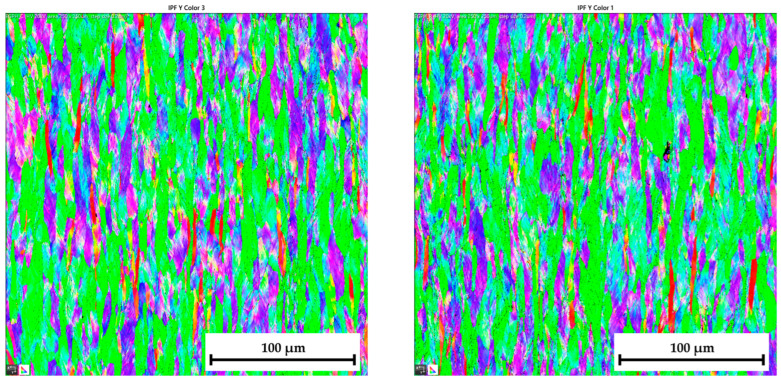
CR4 after deformation—IPF analysis of grain orientation in the Y-direction (**left**—continuous measurement, **right**—ramp test).

Similar to the initial structure, the microstructures after deformation were analysed by EBSD and IPF methods as well. Since the major aim of this paper was to evaluate the changes in the deformation behaviour during the continuous measurement and ramp tests at HBT, the final microstructures are always compared side by side. The resulting microstructures after deformation for both tested materials are shown in the following figures—for DP500, in Figure 25 and Figure 26 and, for CR4, in Figure 27 and Figure 28.

## 4. Discussion

Most stress–strain curves in materials testing are determined by the static tensile test. However, for a reliable description of deformation behaviour, they must be also determined under an equi-biaxial stress state, which is essential, e.g., for the accuracy of the yield conditions used in FEA. These biaxial stress–strain curves are currently measured mainly by contactless optical analysis (DIC) according to EN ISO 16808, where pressure loading is continuously increased during the measurement. However, in the case of the real stamping process, it is sometimes necessary to know the influence of the loading history, which may affect the resulting position of the biaxial stress–strain curves. One of these parameters may be, e.g., the utilisation of holding time under a given loading (e.g., at hydroforming or by using the programable servo-presses).

Therefore, in this work, the effect of such time variation of loading has been investigated at the measurement of the biaxial stress–strain curves using contactless optical deformation analysis. As a reference position for this stress–strain curve (and its subsequent approximation by the Hollomon equation), a common hydraulic bulge test was used, performed in accordance with EN ISO 16808. Because of the subsequent change in loading mode, this test was referred to as a continuous measurement. After that, the influence of holding time at loading was determined as well. To be specific, a holding time of 90 s was applied at the following pressure values: 1; 2; 3; 4; 5; 6; 7; and 7.5 MPa. The actual method of computing the biaxial stress–strain curves was then carried out again in accordance with EN ISO 16808 and these tests were subsequently referred to as ramp tests. The first comparisons of such curves are shown in Figure 9 (DP500) and Figure 15 (CR4).

The first results obtained from the performed tests concerned the change in geometry of the specimen during the holding (dwell) time itself, i.e., within 90 s at each monitored pressure value. In this case, a sphere was first fitted by the best-fit method for the relevant scanned area using the GOM Correlate Pro system to monitor the actual radius of curvature R (mm). The next observed value was always the current maximum position of the sample in the *Z*-axis (thus, height of the dome). Therefore, in the case of the radius of curvature, it was always a certain best approximation within the relevant surface area, while, in the case of the maximum *Z*-axis position value, it was always a single point. The results for the two tested materials are shown in Figure 7 and Figure 8. Based on these results, the deformation behaviour of the tested materials during the holding times can then be explained to some extent. At lower values of pressure (up to about 3 MPa), it is mainly influenced by the change in the radius of curvature so that there is a decrease mainly in the stress values (change in the negative vertical direction within the biaxial stress–strain curve—*Y*-axis). However, there is an increasing influence of the change in the maximum Z position with increasing pressure values, which is observed on the biaxial stress–strain curve by increasing strain magnitude under almost the same stress value (change in the positive horizontal direction + *X*-axis). These increments in terms of ∆R and ∆Z are given in Table 2 and Table 3.

**Figure 29:**
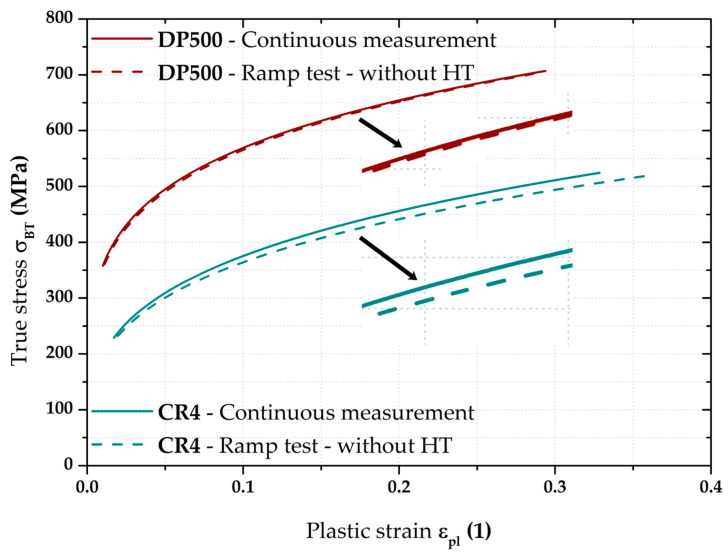
Overview of stress–strain curves for both tested materials.

**Figure 30:**
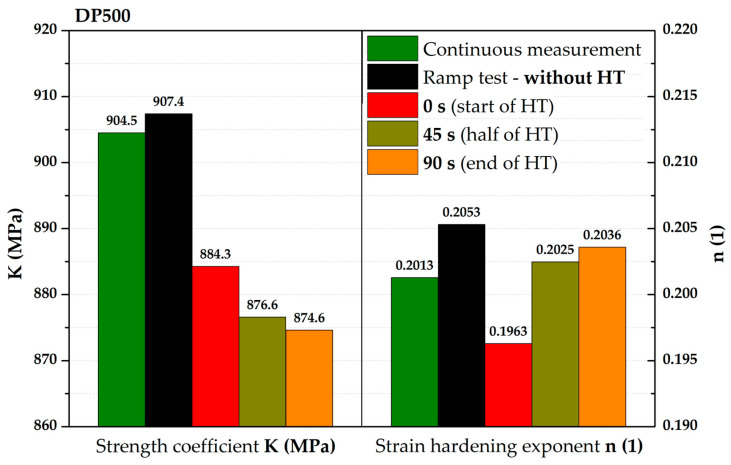
DP500—values of fitting constants (C, n) for all monitored loading modes.

**Figure 31:**
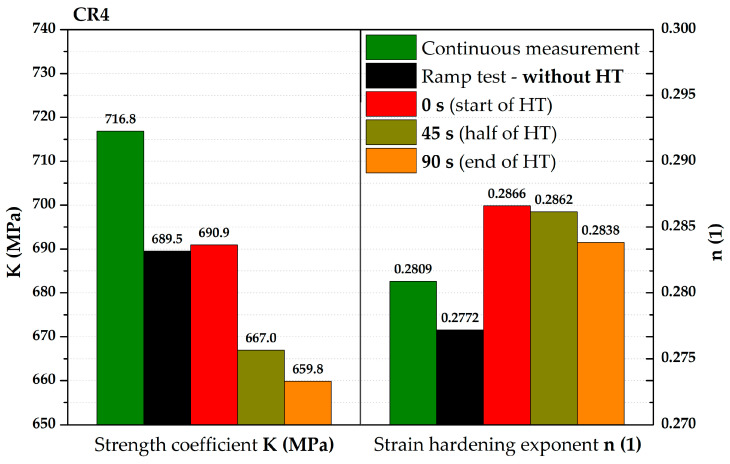
CR4—values of fitting constants (C and n) for all monitored loading modes.

Nevertheless, the main objective of this research was to compare the deformation behaviour of the tested materials in light of continuous measurement and ramp tests. However, it proved useful to divide the ramp test data into several subgroups. Probably the most important approach was to consider only those parts of the biaxial stress–strain curves that were not affected by the regulation and holding time itself. Thus, at this ramp test, only data measured during increasing pressure values were considered, as in the continuous measurement. Graphical examples of this approach are shown in Figure 12 and Figure 18. Here, the different deformation behaviour between the two tested materials is shown. It is clear that the DP500 shows almost no difference between the continuous measurement and the ramp test without the influence of holding times. However, this is not valid for the deep-drawing material CR4, where the difference between the loading methods is evident. These results are subsequently graphically shown in Figures 30 and 31. However, much more clearly and for both tested materials together, these results are shown in Figure 29. The different deformation behaviour regarding the influence of loading mode is evident.

On the other hand, for DP500, it can be seen that the HT at a given pressure value has almost no effect on the subsequent deformation behaviour of this material at the equi-biaxial stress state. It is obvious that two monitored stress–strain curves practically overlap. In contrast, in the case of deep-drawing material CR4, a decrease in the strength coefficient value can be observed. So, in this case, the resulting stress–strain curve for the ramp test without the influence of HT is below the values measured in continuous measurement. For clarity, the values of all fitting constants are shown in Table 6.

Moreover, in addition to such basic comparison between continuous measurement and ramp test without the influence of HT, the influence of the course of HT was also evaluated. More precisely, the effect of the actual time course of the holding time was monitored using three selected time moments—0 s (start), 45 s (half), and 90 s (end) of HT. It is interesting to note that the same time intervals do not have the same effect on the strain response. To describe this effect more accurately, these points were then also approximated using the Hollomon equation, with graphical dependencies shown in Figure 14 and Figure 20. The final values of the fitting constants of all performed analyses are summarised in Table 6.

**Table 6:** Final overview of the fitting constants.

Material/ Measuring Mode	DP500		CR4	
	K (MPa)	n (1)	K (MPa)	n (1)
**Continuous measurement**	**904.5**	**0.2013**	**716.8**	**0.2809**
**Ramp test—without HT**	**907.4**	**0.2053**	**689.5**	**0.2770**
0 s (start of HT)	884.3	0.1963	690.9	0.2866
45 s (half of HT)	876.6	0.2025	667.0	0.2862
90 s (end of HT)	874.6	0.2036	659.8	0.2838

Graphically, these results for material DP500 are shown in Figure 30. If the values obtained from the continuous measurement are taken as a basis (100%), it is interesting to note that the change in the magnitude of strain hardening exponent is not so significant depending on the used loading and evaluation modes and almost all the values (with the minor exception of the start of HT, where there is a decrease of 2.5%) are within a maximum change of 2%. The situation is slightly different for the strength coefficient. The ramp test itself, without the effect of holding times, is almost the same as the continuous measurement (change + 0.3%), while the effect of HT itself is reflected in a gradual decrease in these values up to −3.3% at the end of HT.

The same graphical overview of such major results, in this case for the deep-drawing material CR4, is shown in Figure 31. The values given in Table 6 can be again used for a quick comparison of changes that have occurred under the influence of applying the different loading methods to the material under test. Again, the values of the fitting constants (C and n) obtained from the continuous measurement can be used as reference ones. The most important comparison is again between the continuous measurement and ramp test without HT. There is a decrease of up to 3.8% for the strength coefficient and 1.3% for the strain hardening exponent. A significant decrease in the strength coefficients can also be observed for the time moments during HT—start, half, and end. Specifically, these decreases are as follows: 3.6%; 6.9%; and 8.0%. Regarding the strain hardening exponents, these values then increase slightly by 2.0%, 1.9%, and 1.0%, respectively.

The results described above regarding the effect of loading mode on the final position of stress–strain curves for both tested materials are probably the most important results of this research. It can be seen that there is a relatively large difference between the influence of used loading mode on the deformation behaviour of high-strength steel DP 500 and deep-drawing material CR4. For the DP500 material, it turns out that the application of the holding times has almost no influence on the resulting stress–strain curve, more precisely, its comparison just with the continuous measurement. In contrast, in this comparison, the CR4 material shows a notable decrease in the final stress–strain curve. Numerically, these trends are then evaluated by the Hollomon equation, particularly concerning the resulting strength coefficient values. However, such different deformation behaviour was already observable not only using the changes in radius of curvature R and the maximal dZ position (see chapter 3.2) but also with respect to the results of the strain rates—see chapter 3.1. From this measurement, it can be seen that, in the case of DP500, the strain rate value decreases towards zero, even at higher pressures—already at 7 MPa. On the other hand, in the case of CR4, it can be seen that almost from 5 MPa, the strain rate does not drop to zero during the holding time and, therefore, deformation is still spreading. The higher the applied pressure, the higher the values of strain rate at the end of the applied holding time for the material CR4.

To analyse the microstructure of the tested material before and after deformation, EBSD and IPF analyses were carried out via scanning electron microscope. Due to the HBT, for both materials, the formation of deformation texture was observed. However, already from this measurement, it was evident that a higher directionality was found for material CR4. Moreover, it can be seen that there is almost not any effect of loading mode on the grain size of DP500, which is not valid for material CR4, where differences can be observed in grain sizes concerning used loading mode. It seems that the ferritic-martensitic structure of DP500 can greatly reduce the motion of a dislocation, while the pure ferritic structure of CR4 allows the dislocations to reach to some extent the equilibrium state during the holding time, which results in a reduction in its strengthening. However, this fact was not investigated further in this paper and it would be appropriate to carry out separate material research in this area.

**Figure 32:**
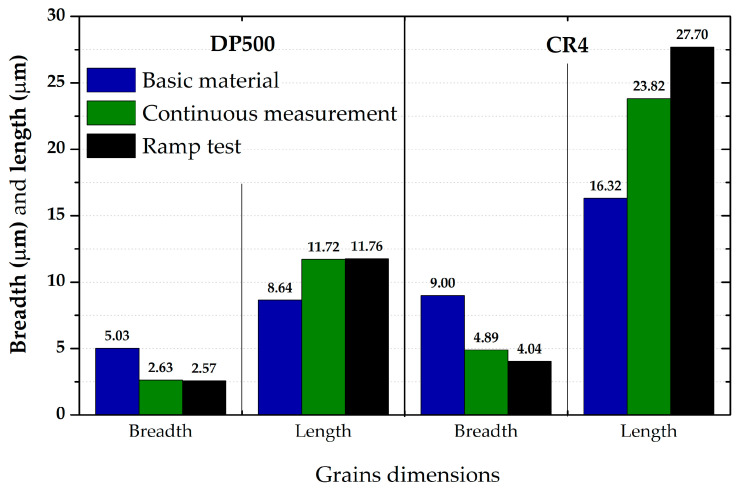
Grain dimensions for all monitored loading modes and tested materials.

From SEM figures, the formation of deformation texture caused by the biaxial stress state at HBT is evident. In order to evaluate the intensity of plastic deformation, which is in the case of HBT determined by the magnitude of plastic deformation in the thickness direction, it is useful to observe the grain sizes after deformation. A general overview of the important grain dimensions (breadth × length) both before and after deformation as well as the used loading mode (continuous measurement and ramp test) is shown in Table 7. A graphical illustration of these results is given in Figure 32.

Table 8 shows percentage representations of individual grain orientations of tested materials. It is again evident that initial structures have typical grain orientations corresponding to the rolling technology. Material DP500 has a significantly lower grain orientation related to the rolling direction and the percentage representation of each direction is almost uniform in this case. The initial state of grain orientation for material CR4 is significantly anisotropic, with a predominant orientation of (111) in the direction of the material thickness (X-direction)—up to 82%. The different deformation behaviours of these two tested materials arises from their different structures (ferritic-martensitic structure of DP500 and pure ferritic structure of CR4). As can be seen also from Table 8, in the case of material DP500 after deformation, there is only a minimal change in the orientation of the crystal lattice caused by the blocking motion of dislocation by martensite grains. On the other hand, in the case of the CR4 material, the pure ferritic structure does not prevent such motion of dislocations in the predominant directions and, therefore, deformation of the grains occurs only and not their rotation. From Table 8, it can be seen that, for material DP500, the loading mode does not affect the resulting percentage representation of grains with (111) orientation. In contrast, in the case of material CR4, it can be seen that there is a further increase in this proportion of grain orientation after the ramp test. These results confirm the different deformation behaviour of both tested materials at HBT when comparing continuous measurements and ramp tests.

From Table 7 and Table 8, it is evident that, in the case of material DP500, there is almost no difference in grain size for both loading modes used (continuous measurement and ramp test). The grain shape and size are almost identical. On the other hand, for material CR4, it is possible to observe the effect of used loading mode on the grain size after deformation, where the ramp test revealed a higher plastic deformation of grains, which is consistent with the measured deformation behaviour during determination of the stress–strain curves. For the material CR4, a secondary plastic deformation (plastic creep) occurs during the holding time under the given pressure loading. This phenomenon can be explained by the different structures of tested materials, where, in DP500, the dislocation motion is resisted by the martensitic grains combined with a finer structure. In comparison, material CR4 has a purely ferritic structure, so dislocation motions to achieve the equilibrium state during the holding time are not limited. This phenomenon, observed in the submitted research, is especially important for numerical simulation of low strain rate technological processes, such as hydroforming.

It is important to note that only two materials (DP500 and CR4) with different deformation behaviour were tested in this paper. In addition, due to the considerable time and hardware requirements of the measurements, especially in the case of ramp tests, five specimens were always used for each tested material to obtain biaxial stress–strain curves. Further research in this area is currently focused on obtaining more material data to assist in the necessary verification of the presented results. On the other hand, this paper has already shown a relatively good agreement with the already made measurements. Much attention is also being given to the use of these results as input data for numerical simulations of sheet metal forming and their verification on real stampings.

Given the planned further research about the influence of loading modes on the biaxial stress–strain curves, it would be advisable to test specimens also of other important material groups, such as stainless steels or aluminium alloys. Based on the measurements made so far, the length of HT also appears to be an important characteristic, as it has already been shown in this paper that this is certainly not a directly proportional dependence. The deformation behaviour over the time course of these HTs was also somewhat different for the two materials. However, to monitor the influence of the various parameters on the test presented, it would be useful to also consider, e.g., PID control settings for decelerating and accelerating the time change of pressure around HT, the influence of strain rate, or, for example, the grain size of used materials.

## 5. Conclusions

The main objective of the present research was to investigate the effect of time change of loading mode on the equi-biaxial loading of tested materials. Specifically, it was a comparison of the final position of the biaxial stress–strain curve when measured in accordance with ČSN EN ISO 16808 (i.e., with a continuous time increase in pressure values until material failure) with the biaxial stress–strain curve when a holding time of 90 s was applied at selected pressure values. So, the first step was to determine the biaxial stress–strain curves by continuous measurement, followed by so-called ramp tests. The intention was not only to observe the effect of these holding times themselves but also to determine the effect of their time course. For this reason, the results of the ramp tests were then divided into several subgroups. Specifically, the influence of these HT was monitored when considering only increasing pressure, as well as start, half, and end points of HT.

The test materials were deliberately chosen to represent two different groups of materials. In this case, a two-phase high-strength steel DP500 and a deep-drawing material CR4 were used. From a material point of view, this was a comparison of the deformation behaviour of a martensitic-ferritic structure with a purely ferritic structure. In this case, the GOM Correlate Pro system was chosen to perform the contactless optical deformation analysis. The final measured biaxial stress–strain curves were then approximated using the Hollomon equation. The resulting positions of these curves and the values of the fitting constants were used to compare measured data. The following conclusions can be stated from the measured and computed results:

- Ramp tests at HBT are a very time-consuming and hardware-intensive kind of testing;
- Different deformation behaviours of tested materials can be already observed by the strain rate values during the holding times;
- During the holding time, the influence of change in the radius of curvature is greatest at lower pressures, while the effect of the maximal dZ position (height of the dome) becomes increasingly apparent as the pressure value increases during the HT;
- It is very useful to divide the results of the ramp tests into several subgroups and then to monitor the resulting biaxial stress–strain curves;
- Major differences between the tested materials were monitored via comparison of the final stress–strain curves from the continuous measurement and ramp test without the influence of HT, where DP500 was not influenced by the loading mode, while CR4 was;
- In light of the course of holding time (totally 90 s), the largest changes occurred during the first 45 s, but the influence of the second half of HT (from 46 to 90 s) increased with increasing pressure value;
- Dislocation motion is more strongly resisted in the ferritic-martensitic structure of DP500 compared to the pure ferritic structure of CR4;
- From the grain dimensions after deformation point of view, there is almost no difference between continuous measurement and ramp test for DP500, but this difference can be observed in the case of CR4;
- It should be very useful to test also other material groups (e.g., aluminium alloys) as well as to determine the influence of grain dimensions or strain rates in measuring HBT under different loading modes.

## Figures and Tables

**Table 7:** Overview of grain dimensions for all monitored loading modes and tested materials.

Material	DP500		CR4	
**Grain dimensions**	breadth (µm)	length (µm)	breadth (µm)	length (µm)
**Basic material**	5.03	8.64	9.00	16.32
**Continuous measurement**	2.63	11.72	4.89	23.82
**Ramp test**	2.57	11.76	4.04	27.70

**Table 8:** Percentage representation of grain orientation in the X-direction (thickness direction).

Material	DP500			CR4		
**Grain orientation (%)**	Red	Green	Blue	Red	Green	Blue
**Basic material**	28.7	12.1	36.2	10.2	4	82
**Continuous measurement**	34.2	3.1	39.2	10.3	1.2	87.1
**Ramp test**	33.7	2.8	40.4	7.1	0.7	92

## Data Availability

The data presented in this study are available on request from the corresponding author due to legal or ethical reasons.
